# One size may not fit all: anti-aging therapies and sarcopenia

**DOI:** 10.18632/aging.100409

**Published:** 2011-12-16

**Authors:** Tyesha N. Burks, Ronald D. Cohn

**Affiliations:** ^1^ McKusick-Nathans Institute of Genetic Medicine, Johns Hopkins University School of Medicine, Baltimore, MD 21205, USA; ^2^ Department of Pediatrics and Neurology, Johns Hopkins University School of Medicine, Baltimore, MD 21205, USA

**Keywords:** sarcopenia, therapy, caloric restriction, caloric restriction mimetics, anti-aging, skeletal muscle

## Abstract

Sarcopenia refers to age-related loss of muscle mass and function. Several age-related changes occur in skeletal muscle including a decrease in myofiber size and number and a diminished ability of satellite cells to activate and proliferate upon injury leading to impaired muscle remodeling. Although the molecular mechanisms underlying sarcopenia are unknown, it is tempting to hypothesize that interplay between biological and environmental factors cooperate in a positive feedback cycle contributing to the progression of sarcopenia. Indeed many essential biological mechanisms such as apoptosis and autophagy and critical signaling pathways involved in skeletal muscle homeostasis are altered during aging and have been linked to loss of muscle mass. Moreover, the environmental effects of the sedentary lifestyle of older people further promote and contribute the loss of muscle mass. There are currently no widely accepted therapeutic strategies to halt or reverse the progression of sarcopenia. Caloric restriction has been shown to be beneficial as a sarcopenia and aging antagonist. Such results have made the search for caloric restriction mimetics (CRM) a priority. However given the mechanisms of action, some of the currently investigated CRMs may not combat sarcopenia. Thus, sarcopenia may represent a unique phenotypic feature of aging that requires specific and individually tailored therapeutic strategies.

## INTRODUCTION

Aging is a multi-factorial process affecting every organ of the body. In the past, aging research has primarily focused on neurodegeneration and loss of bone mass. Not much attention has been given to sarcopenia until recently. Sarcopenia refers to the physiological loss of skeletal muscle mass and function during aging [1]. Several age-related changes occur in skeletal muscle including a decrease in myofiber size and number and a diminished ability of satellite cells to activate and proliferate upon injury leading to impaired muscle remodeling [2, 3]. The progressive loss of muscle mass poses health risks for older adults that lead to a decrease in physical activity and a rise in the incidence of falls and related fractures. Rehabilitation time is often prolonged after injury, which in turn extends the duration of bed rest resulting in disuse atrophy, an additional variable that is exaggerated in the aging population [4-6] thus interfering with a successful recovery.

Sarcopenia is a major public health problem affecting approximately 25% of people under the age of 70 and 40% of people age 80 years and older [7]. In 2000, sarcopenia-related healthcare expenses totaled approximately $18.5 billion in the United States [8]. However, the projected aging-related expenses are expected to exponentially increase because older people are the fastest growing population in the US [9] with the number of individuals over 60 years of age doubling over the next 40 years [10].

Considering the impact of sarcopenia on the well-being of older adults and the healthcare system in general, it is critical to identify widespread therapeutic strategies to maintain skeletal muscle homeostasis and repair. While strength and aerobic exercise have been documented to attenuate [11] and even reverse sarcopenia [12, 13], a recent study reported only 2% of older subjects exercised on a regular basis [14]. Therefore, there is a great need for a more prevalent intervention to combat sarcopenia, in particular one that accounts for the interplay of biological and environmental factors would be a preferred because they both contribute to the vicious cycle that drives sarcopenia. Recent studies have highlighted caloric restriction (CR) and caloric restriction mimetics (CRM) as potential mechanisms to combat aging and sarcopenia. This article summarizes the current knowledge about the effects of these therapies on aging skeletal muscle.

### Biological Factors

The molecular mechanisms underlying sarcopenia are not fully elucidated. However several lines of evidence have connected many different age-related changes to the resulting decrease in muscle mass and function: increases in apoptosis and reactive oxidative species (ROS); decreases in autophagy; modulation of signaling pathways involved in skeletal muscle homeostasis and excitation-contraction (EC) uncoupling. All of these factors appear to contribute to the progression of sarcopenia albeit no single factor can independently account for the aging-related changes occurring in skeletal muscle.

#### Factors Impacting Skeletal Muscle Structure

Histologically, the age-related loss of muscle mass is characterized by a decrease in muscle fiber size and number with a preferentially loss of type II fibers [15, 16]. Due to the impaired ability of skeletal muscle to remodel, the loss of myofibers can be accompanied by the infiltration of adipose tissue, inflammation and/or fibrotic tissue [17-21]. Other markers of impaired remodeling such as variation of fiber size, increase in centralized nuclei and an increase in atrophic, angulated fibers are often present [22]. Proposed mechanisms for the loss of muscle size and number including increased ROS, mitochondrial DNA (mtDNA) mutations, impaired autophagy and increased apoptosis appear to function synergistically to contribute to the pathogenesis of sarcopenia.

A progressive decline in mitochondria function leads to an increase in the formation of oxidative stress and ROS production. The increase in ROS leads to mtDNA deletions and mutations, macromolecular oxidation, electron transport chain (ETC) dysfunction, cellular senescence and cell death [23-25]. Oxidative damage to proteins may alter their structure and function leading to the formation of aggregates. Oxidized proteins may be ubiquitinated in order to be targeted by the proteosome for degradation. However during aging of skeletal muscle, there is an increase in the accumulation of oxidized proteins, attributed to a decline in the activity of the proteosome [26]. Conversely, recent data report an increase in the number of proteosomes with normal degradative capacity, suggesting enhanced proteosome activity contributes to muscle loss [27]. Therefore, the role of the ubiquitin-proteosome activity in sarcopenia requires further clarification. Nonetheless under normal physiological conditions, the cell undergoes autophagy to protect itself from accumulation of protein aggregates, but there is a decline in the activity and efficiency of autophagy with age. Consequently there is an intracellular accumulation of protein aggregates, impaired autophagy [28], increased apoptosis and oxidative stress [28] associated with impaired energy production due to damaged mitochondria [28-30].

With age, alterations in the expression of autophagy-related genes have been reported. Studies have shown an increase in Beclin-1 and a decrease in LC3, LAMP-2, Bnip-3 and Gabarap1 [28, 31]. These changes lead to impaired autophagy with a decline in its degradation activity causing post-mitotic cells to accumulate biological garbage. Furthermore, the process of autophagy is necessary to maintain skeletal muscle mass. Skeletal muscle specific inhibition of autophagy using mice deficient in Atg7 has resulted in a phenotype similar to sarcopenia: muscle atrophy, loss of force, accumulation of abnormal mitochondria, protein aggregates, oxidative stress and apoptosis [32, 33].

Accelerated apoptosis, as seen in aging, likely also contributes to the loss of myofibers observed in sarcopenia. Apoptosis could be a consequence of the impaired autophagy and abnormal mitochondria. Studies show an increase in mRNA, protein and/or activity levels of many pro-apoptotic markers including the BCL-2 family, caspases, Apaf-1, XIAP and cytochrome c [34, 35]. These increases are accompanied by an increase in apoptotic DNA fragmentation [28, 34] and a compensatory up-regulation of anti-apoptotic factors [35-37]. Due to the multinucleated nature of skeletal muscle, it can undergo individual myonuclear apoptosis or complete cell death. The phenomenon of myonuclear apoptosis supports the nuclear domain hypothesis that states a single nucleus controls a defined cytoplasmic area. Under this hypothesis, the removal of myonuclei is necessary for muscle atrophy to occur [6, 38].

Age-related changes also occur in a number of critical signaling pathways that further promote the process of aging. Although there are several signaling pathways linked to sarcopenia, we focus our discussion on the canonical and non-canonical transforming growth factor-β (TGF-β) cascade, Wnt signaling and the insulin-like growth factor-1 (IGF-1)/Akt/mammalian target of rapamycin (mTOR) pathways.

Loss of muscle mass in sarcopenia has been linked to the modulation of the canonical (Smad-dependent) and non-canonical (Smad-independent) TGF-β signaling cascades [3]. An increase in circulating TGF-β1 and phosphorylated Smad3 levels contribute to the formation of connective tissue within the extracellular matrix (ECM). This interferes with the satellite cell niche, creating an environment that inhibits satellite cell activation and proliferation [3, 39], impairs myocyte differentiation [39, 40] and leads to the formation of fibrotic tissue in response to skeletal muscle injury [41]. There are conflicting data concerning the activation of the non-canonical TGF-β mitogen-activated protein kinase (MAPK) pathway; it has been reported to be up-regulated, down-regulated and unchanged in aged skeletal muscle [5, 42, 43]. Furthermore, MAPK has been shown to participate in a variety of functions including muscle regeneration, remodeling, and contractions.

Although the exact role and concentrations of MAPK signaling in sarcopenia requires more research, it has been associated with creating a ‘stress-like’ condition in which aged muscle is constantly exposed when up-regulated [42] and contributing to the impaired regeneration [5].

Wnt signaling has been shown to be involved in satellite cell proliferation and differentiation in skeletal muscle regeneration [44, 45]. It has been suggested that a temporal switch from notch to Wnt signaling occurs during the onset of differentiation [44], suggesting that timing and tissue homeostasis of these signaling pathways are important for efficient regeneration. Therefore impaired regeneration in aged skeletal muscle could be associated with an aging-related increase in the activation of Wnt signaling, evident by increased levels of Axin7 and β-catenin and a decrease in phosphorylated GSK3β in non-injured aged skeletal muscle cells and satellite cells and myogenic progenitor cells upon injury [44, 46]. Furthermore, the sustained activation of Wnt signaling in skeletal muscle is associated with stem cell aging and the transformation of myogenic to fibrotic tissue [46].

IGF-1 is a growth factor whose activation is critical in mediating the growth of skeletal muscle and its levels decrease with age [47]. Several lines of evidence have shown that the local administration of IGF-1 directly into skeletal muscle prevents the age-related loss of muscle mass [48], function [47] and regeneration [48]. Akt is a downstream effector of the IGF-1 pathway. Akt can induce protein synthesis through the phosphorylation and activation of mTOR and inhibit protein degradation through the phosphorylation and inactivation of FoxO3a. mTOR signaling is critical for muscle homeostasis, all stages of regeneration [49-51] and muscle hypertrophy [52-54]. FoxO3a signaling induces the transcriptional activation of atrogenes and autophagy-related genes resulting in protein degradation. Interestingly, there are discrepancies in the expression levels of atrogenes, MuRF-1 and atrogin-1, in sarcopenia [31, 55, 56]; although they have been linked with acute conditions of muscle atrophy [56, 57]. Thus, this pathway likely does not play a primary role in sarcopenia because its modulation depends on sex, age and muscle fiber type [58]. However, it is well documented that the loss of muscle mass during disuse as the result of inactivity and bed rest in young and aged skeletal muscle is associated with a reduction in the Akt/mTOR pathway [59-61]. Notably, sarcopenic muscle lacks the ability to sufficiently recover from disuse-induced atrophy as compared to young muscle [62]. Taken together, the existing data suggest that the IGF-1/Akt/mTOR pathway does not play a primary role in the process of sarcopenia, and that other regulators will need to be identified as possible modulators of sarcopenia.

#### Factors Impacting Skeletal Muscle Function

As a consequence to the loss of skeletal muscle mass, there is a 20-40% decline in muscle function [63]. This decline in function has been linked to several factors including the loss of muscle mass, apoptosis and impaired energy production due to damaged mitochondria as discussed above. However, none of those factors have sufficiently accounted for the decline in muscle function. Evidence has shown that the Ca^2+^ dependent EC coupling process is impaired with age and contributes to the decline in function [64].

Studies have shown that there is a decrease in the number of dihydropyridine receptors (DHPR) resulting in an increase in the amount of DHPR-unlinked ryanodine receptors (RyR1) during aging. Therefore EC uncoupling occurs and results in impairment of the voltage-gated SR Ca^2+^ release mechanism and a decline in contraction force [65]. Furthermore, the increase in oxidative stress causes nitrosylation and oxidation of the RyR1 complex. The defective RyR1 complex causes an intracellular Ca^2+^ leak that further disrupts mitochondria structure and function [66]. EC uncoupling and leaky RyR1 likely contribute to the loss of muscle function in sarcopenia.

### Environmental (lifestyle) Factors

#### Nutrition

Preservation of skeletal muscle mass is achieved by maintaining a homeostatic balance between protein synthesis and degradation [67]. During aging there is impaired protein metabolism associated with a decline in total food intake (anorexia of aging) [68]. This leads to deprivation of amino acids which blocks protein synthesis in older individuals. Furthermore, the anorexia of aging may lead to malnutrition, which is linked to modulation of different hormones including testosterone, leptin, growth hormone, and IGF-1 that contribute to muscle wasting [69].

#### Activity

The decrease in muscle mass and strength has significant functional consequences for the elderly population. Functional impairment defined as difficulty in mobility performance including walking, stooping and standing up from a chair and physical disability defined as difficulty with performing daily activities such as chores and cooking occurs 2 times and 3 times more likely in men and women, respectively, with sarcopenia when compared to those considered to have normal skeletal muscle index [14].

The decline in physical activity increases susceptibility to disuse atrophy, a frequent problem for individuals of all ages, but a particularly challenging one for older adults. When skeletal muscle is subjected to disuse for a prolonged period of time (i.e. bed rest), muscle atrophy occurs [1]. This atrophic response is a completely reversible process in the younger population [1]; however as a result of the physiological process of aging, humans are known to exhibit an exaggerated atrophy in response to disuse and an inability to rebuild muscle mass after immobilization [62, 70]. Studies performed in human subjects reported a 30% loss of skeletal muscle mass after only two weeks of immobilization in older men as compared to a loss of less than 2% in young men, and only 2.5% of the loss muscle repopulated [5]. Research has shown that immobilization in aged subjects leads to a loss of muscle cells as opposed to smaller cells observed in young muscle [60, 71].

#### Injury

Advanced age, muscle weakness, lack of physical activity and functional limitations are risk factors that increase the incidence of falls and related fractures in older persons. Twenty to thirty percent of elderly people who fall sustain injuries that further reduce mobility and independence, thereby increasing the sedentary lifestyle and bed rest [9]. Furthermore, the damage to the skeletal muscle upon fractures and the impaired regeneration due to modulation of signaling pathways leads to the replacement of skeletal muscle with connective tissue and a further decline in function and activity [60].

### Therapeutic Interventions: Aging-focused

**Figure 1:**
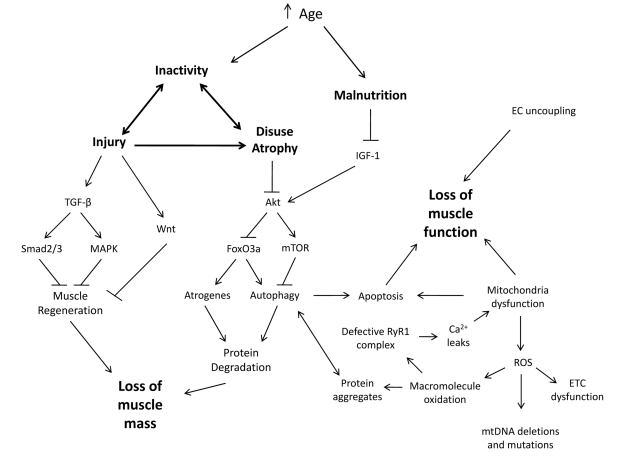
Synergistic Interplay between Biological and Environmental Factors Contribute to Sarcopenia With an increase in age, people are subjected to environmental changes such as inactivity and malnutrition which leads to an increased susceptibility to injury and disuse atrophy. When skeletal muscles incur such challenges, alterations to signaling pathways promote inefficient muscle regeneration and protein degradation resulting in a loss of muscle mass. This loss of muscle mass and other biological changes are concurrent with a loss of muscle function.

The pathogenic etiology of sarcopenia is complex, characterized by the contribution of multiple factors [72]. It is reasonable to hypothesize that both biological and environmental factors contribute to its progression (Figure 1). Due to its multi-factorial nature, researchers have taken different avenues to modulate phenotypes to combat sarcopenia. Recent advances in anti-aging regimens have generated interest in the ability of these strategies to slow the progression of sarcopenia. Despite the complexity of aging and sarcopenia, it has been hypothesized that the anti-aging regimens should also benefit sarcopenia.

#### Caloric Restriction

One intervention that has been shown to attenuate sarcopenia is CR, the reduction in caloric intake without malnutrition [73]. The effects of CR on sarcopenia have been extensively studied using different model organisms, skeletal muscle groups and various dietary restrictions [30, 74-76]. It slowed down the progression of sarcopenia by decreasing the amount of myofiber atrophy and loss [75-78]. It also maintained the specific force and size of type II fibers [21]. Furthermore, it attenuated skeletal muscle remodeling associated with sarcopenia by decreasing the amount of fibrotic tissue, variation in fiber size, centralized nuclei and angulated fibers [21, 22]. This phenotype was achieved through the modulation of different biological factors associated with sarcopenia.

The reduction of caloric intake alleviated the burden of ROS-related consequences by reducing oxidative damage sustained to the mitochondrial proteins and lipids, the generation of superoxide anions [79], and the accumulation of deleted mitochondrial genomes and mitochondrial enzyme abnormities with age [75]. It also prevented the decline in autophagy by increasing the expression of Atg proteins and LC3 and LAMP2 genes [28]. The trypsin-like proteosome activity was sustained but did not result in a decrease of protein aggregates [26]. Furthermore, the increase in apoptosis was attenuated by a reduction in the levels of intrinsic and extrinsic apoptotic signals including the caspases, XIAP and AIF [35, 36, 80]. CR also prevented the age-related decline in muscle function [81] by maintaining the ratio of DHPR and RyR1 and preventing uncoupling [82].

CR has been proven to correct multiple detrimental effects of aging on skeletal muscle as well as other age-related diseases and longevity. However, implementing CR as a valuable therapeutic avenue for sarcopenia and aging consists of numerous problems [83]. One particular problem is determining the exact time frame for starting CR. When started too early in life, it may cause developmental problems and if started too late, benefits may not be achieved [84]. Furthermore, CR, started late in life could contribute to the anorexia of aging and mortality [85]. Nonetheless the benefits of CR on aging and sarcopenia have lead to the search for CRMs.

#### Caloric Restriction Mimetics

CRMs are compounds that allow individuals to eat ad libitum while benefitting from the effects of CR. Ingram et al. proposes that potential CRM should meet the following criteria: (i) it mimics the metabolic, hormonal, and physiological effects of CR; (ii) it does not significantly reduce long-term food intake; (iii) it activates stress response pathways observed in CR and provides protection against a variety of stressors; and (iv) it produces CR-like effects on longevity, reduction of age-related disease and maintenance of function [86].

There are four main pathways that are the target for the development of CRMs: insulin/insulin-like growth factor 1 (IGF-1), sirtuin 1 (SIRT1), target of rapamycin (TOR), and 5' adenosine monophosphate-activated protein kinase (AMPK) [87]. The majority of these pathways have been shown to extend lifespan and health span through pharmacological and genetic manipulation cite [88-91]. Common compounds used to manipulate these pathways include metformin, resveratrol and rapamycin.

Metformin is a biguanide drug used to treat type-2 diabetes, but the primary mechanism of hypoglycemic action is unknown [92]. However, it has been shown to inhibit the complex 1 of respiratory chain complex of the mitochondria as well as causing an increase in peripheral glucose utilization in skeletal muscle [93]. Evidence has shown that metformin does not cause glucose uptake in skeletal muscle in non-diabetics [94, 95]. Despite the conflicting information on the ability of metformin to extend lifespan [96, 97], it is a candidate CRM. It is proposed to affect the AMPK, sirtuins, and TOR pathways [98]. Whether metformin is a valuable target for sarcopenia is questionable. In particular, its inhibitory effect of the mTORC1 signaling [99] may interfere with myogenesis and maintenance of muscle mass.

Resveratrol is a small polyphenol in fruits and red wines at low concentrations [100]. It has been suggested to exhibit antioxidant and cardioprotective properties [100]. It has been identified as a CRM due to its potential ability to increase SIRT1 protein levels [101]. However, reports have shown that it may not directly up-regulate SIRT [102] and that its exact molecular mechanism is unknown. Resveratrol is known to activate and inhibit many different enzymes including AMPK [103], which is another pharmacological target for CR [104, 105]. Given the ability of resveratrol to either directly or indirectly affect sirtuins, AMPK and autophagy [103, 106, 107], it seems like a promising candidate to treat sarcopenia. Moreover, resveratrol was shown to be protective against oxidative stress associated with loading, unloading and aging in skeletal muscle [106, 108]. However, it did not attenuate the age-related decline in muscle mass or function associated with sarcopenia in rodents over a prolonged period of time. Furthermore, resveratrol did not affect the levels of SIRT1, PGC-1α, or cytochrome C in aged skeletal muscle [109].

Rapamycin, an immunosuppressant that may have adverse effects in healthy individuals [110], inhibits TOR signaling [88]. Increased mammalian TOR (mTOR) signaling is a hallmark of the aging phenotype and mTOR-centric views of aging have recently emerged [111]. Studies have shown that inhibition of mTOR signaling is associated with ameliorating several age-related phenotypes including decelerating cellular senescence, altered translational control, increased mitochondria number and cellular respiration [112-114]. Moreover, researchers have shown that mTOR may actually promote autophagy in a TORC Autophagy Secretory Colocalization Compartment (TASCC) in senescence cells as opposed to inhibiting autophagy outside out of the TASCC [115]. This has lead to the hypothesis that TOR signaling is alternatively regulated during aging. Indeed, it is known that TOR signaling has tissue-specific functions in mammals (71). Perhaps systemically inhibiting TOR may prove problematic to cellular functions including secretory protein autophagy and to organs including skeletal muscle [110]. With age in humans, there is a decrease in TOR signaling in muscle [52] that may be linked to the progression of sarcopenia because TOR signaling is important in skeletal muscle homeostasis, all the stages of regeneration [49-51] and muscle hypertrophy [52-54]. Therefore, systemically treating organisms with rapamycin may impair the muscles' ability to regenerate upon injury and hypertrophy following exercise. Furthermore, rapamycin was shown to cause a loss of EC coupling [116] and a reduction in voltage-gated Ca2+ release [117] contributing to functional decline. Detailed pre-clinical studies in rodents are necessary to evaluate any potential negative side effects of TOR inhibition on skeletal muscle mass and function.

### Therapeutic Interventions: Skeletal muscle-focused

The multiple benefits of CR, not only on sarcopenia but also on other age-related diseases, has supported the search for and use of CRMs to combat aging and extend longevity. However the current CRMs do not elicit such a widespread effect as CR itself and there is insufficient evidence that they are able to combat sarcopenia. It is therefore important to entertain the hypothesis that management of sarcopenia may consist of muscle-specific regimens that may be used in conjunction with systemic anti-aging treatments.

#### Nutrition and Exercise

Studies have shown that supplementing nutritional intake with amino acids spares skeletal muscle from sarcopenia. It causes an increase in mTOR signaling believed to result in increased cross-sectional area of myofibers and protein synthesis [54, 67]. Furthermore, it restored the ratio of type I and 2A fibers and increased sarcomere volume [67]. Another contributing factor to sarcopenia is a decline in physical activity and increased susceptibility to disuse atrophy. For decades, exercise has been recommended to slow down sarcopenia. In humans, high-intensity resistance training increased muscle strength and cross-sectional area [11, 12]. Furthermore, it is proposed that the combination of increased amino acid intake and daily physical activity can additively combat sarcopenia [54]. Although exercise and nutritional intake are the current strategy for managing sarcopenia [118], pharmacological approaches may be necessary given that dietary and exercise regimens are challenging for the elderly.

#### Pharmacological-based

Other strategies aimed at ameliorating sarcopenia directly exploit hormonal imbalances and alterations of signaling pathways critical for skeletal muscle tissue homeostasis. A number of hormones, cardiovascular drugs, anti-inflammatory drugs, and metabolic agents are currently being investigated in regard to a positive effect on skeletal muscle mass and function and potential use to prevent and/or attenuate sarcopenia. We will highlight only a few selected molecules; for an extensive review please see [119].

Testosterone supplementation caused an increase in body and muscle weight attenuating the muscle loss associated with sarcopenia. It increased the CSA of both fiber types and prevented the age-related fiber-type shift [120]. Furthermore, it reduced oxidative stress and apoptosis [120] while increasing the rate of protein synthesis [121] and number of satellite cells [122]. The molecular mechanisms underlying the protection mediated by testosterone included suppression of myostatin and the non-canonical TGF-β pathway via JNK signaling. In addition, testosterone activated Notch1, Akt and G6PDH [120]. The effects of testosterone on aged muscle function are conflicting. While most investigators have reported an increase in muscle strength [121, 123, 124], there is also evidence of no changes [125]. Testosterone treatment is controversial due to its side effects including increased risk of cardiovascular problems and pedal edema. Furthermore, it is not recommended for patients at risk for some medical conditions including sleep apnea, urinary tract symptoms and erythrocytosis [119]. An alternative treatment strategy is the use of synthetic androgen modulators that elicit similar results without the additional health concerns [126-128].

The renin-angiotensin pathway has recently been implicated in the progression of sarcopenia [129, 130]. It increases the production of pro-inflammatory molecules and promotes degradation of muscle proteins. Angiotensin converting enzyme (ACE) inhibitors and angiotensin II receptor blockers (ARBs) are two classes of drugs used to mediate this pathway and both classes have been studied in sarcopenia. Many clinical trials have investigated the use of ACE inhibitors as treatment against sarcopenia with non-uniform functional results; some reports show improvement while others show no change [131-133]. Losartan, an ARB, has shown promising pre-clinical results in the treatment of disuse atrophy and impaired regeneration in the context of sarcopenia through the modulation of the TGF-β and Akt/mTOR pathways [60]. However, its effects on the progression of sarcopenia in humans remain to be elucidated. One pre-clinical study in rats showed an attenuation of muscle strength but did not investigate the muscle morphology [134].

IGF-1 has been linked to different aspects of skeletal muscle homeostasis including growth, differentiation, survival, regeneration and functional profile [47, 135]. It is documented that systemic levels of IGF-1 decrease with age, however, the expression levels of muscle intrinsic IGF-1 are not known. Several investigators have shown that skeletal muscle specific over-expression of IGF-1 is beneficial for sarcopenia. The over-expression of IGF-1 prevents the decline in the number of DHPR and RyR1 and restores the functional capacity of aged mice [136]. Moreover, localized expression of IGF-1 prevented sarcopenia and restored the regenerative capacity of aged skeletal muscle [48]. IGF-1 is beneficial to skeletal muscle; however, its sustained expression in cardiac muscle can lead to pathological hypertrophy [137, 138]. Therefore, it is important to identify molecular targets that increase this growth factor specifically in skeletal muscle.

## DISCUSSION

Sarcopenia is a devastating condition that can lead to disability, increased morbidity and mortality. The age-related modulation of a variety of signaling pathways together with environmental constraints imposed on elderly patients accelerate its progression. The etiology of sarcopenia is linked to a variety of pathogenic mechanisms and it is therefore challenging to identify targeted therapies. The only currently existing management for sarcopenia consists of nutritional supplementation and an exercise regimen, albeit with only mild beneficial effects. However, all geriatric patients cannot benefit from this treatment. Because normal muscle mass and strength are required to perform daily activities, it is imperative to identify pharmacological compounds that can prevent or slow the progression of sarcopenia.

CR has been shown to attenuate various aspects of sarcopenia; however, its primary mechanism of action is unknown. The beneficial results have lead to the search of CRMs but none of these compounds have been demonstrated to combat sarcopenia. Furthermore, it is important to emphasize that some of the pathways modulated by CRMs are critical for skeletal muscle homeostasis casting some doubts as to their potential benefit in sarcopenia. For these reasons, it is imperative to identify tissue-specific alterations and responses to therapies in aging.

The aging of skeletal muscle is unique from other tissues. Skeletal muscle is a post-mitotic, multi-nucleated tissue. Therefore, it is not subjected to cellular senescence with age and can undergo myonuclear apoptosis. Furthermore, skeletal muscle atrophies with age whereas some tissues hypertrophy. Differences also exist in the signaling pathways involved in aging. For example, mTOR signaling is increased in other tissues but decreased in muscle contributing to various age-related pathological phenotypes. These intrinsic differences make finding a “one size fits all” therapy for aging nearly impossible. Thus far, the only treatment that has shown some benefits for various aspects of aging is CR, although detailed molecular knowledge about these benefits are still lacking. Once the molecular mechanisms leading to beneficial results of CR have been further characterized, it will be possible to identify a more specific CRM that emulates the effects of CR. It is therefore necessary to combine multiple therapies with organ-specific benefits while considering potential detrimental effects to other organs.
